# Risk of nonunion for dual plate versus single plate fixation for humeral shaft and distal humerus fractures: a systematic review and meta-analysis

**DOI:** 10.1007/s00590-025-04491-2

**Published:** 2025-08-31

**Authors:** Anthony N. Baumann, Grayson M. Talaski, Shahabeddin Yazdanpanah, Timothy Guthrie, Tyler Sanda, Michael Makowski

**Affiliations:** 1 College of Medicine, Northeast Ohio Medical University, Rootstown, USA; 2 Department of Orthopaedic Surgery, University of Iowa, Iowa City, USA; 3 Department of Orthopaedic Surgery, Cleveland Clinic Akron General, Cleveland, USA; 4 Department of Rehabilitation Services, University Hospitals, Cleveland, USA

**Keywords:** Humerus fractures, Nonunion, Humeral shaft fractures, Distal humerus fractures, Dual plate fixation

## Abstract

**Objective:**

This study’s purpose was to compare clinical outcomes in adult patients who underwent dual plate versus single plate fixation for humerus fractures to enhance decision-making and patient care.

**Methods:**

*Data sources*: A pre-registered systematic review and meta-analysis searched four databases on January 9th, 2025. Inclusion criteria were studies that compared dual versus single plate fixation for humerus fractures.

Outcomes included risk of nonunion, risk of total complications, and time until union after surgery. A random effects meta-analysis was done using risk ratio (RR) or unstandardized mean difference.

**Results:**

Thirteen moderate quality retrospective studies were included out of 211 articles retrieved. Patients (*n* = 788; 47.8% male) had an average age of 50.4 ± 14.2 years and an average follow-up time of 18.1 ± 14.4 months with 378 patients (48.0%) receiving dual plate fixation and 410 patients (52.0%) receiving single plate fixation for humerus fracture (38.4% distal humerus; 61.7% humeral shaft). There was no statistically significant difference in risk of nonunion between dual and single plate fixation groups (*p* = 0.502; RR: 0.99; 5.0% vs.7.3%). For subgroup analysis by location, there was no statistically significant difference in risk of nonunion for distal humerus fractures or humeral shaft fractures, by surgical indication for risk of nonunion for primary fracture or nonunion, or by surgical indication for time until union for primary fracture or nonunion. There was no statistically significant difference in total complications in all subgroups, except dual plate had an increased risk of total complications versus single plate for distal fractures (*p* = 0.014; RR: 1.40).

**Conclusion:**

There may be no clinically meaningful differences in risk of nonunion, risk of total complications, and time until union between dual plate versus single plate fixation for humerus fractures in adult patients.

## Introduction

Humeral shaft and distal humerus fractures represent complex upper extremity fractures in adult patients, with the ideal treatment strategy remaining in debate, depending on a myriad of factors [1–5]. Indeed, the incidence of distal humerus fractures and humeral shaft fractures may be increasing due to an aging population [6], suggesting a growing need for optimized patient care by orthopedic surgeons. Although most humerus fractures can be treated conservatively [7], complications such as nonunion after initial conservative treatment of humerus fractures remain a significant risk with rates as high as 20.6% in humeral shaft fractures [8, 9]. Similarly, nonunion has been reported at rates from 2–21% after surgical fixation of humerus fractures [5], suggesting that nonunion is one of the most common and devastating complications after both surgical and non-surgical management. Among numerous other surgical considerations, successful operative management of humerus fractures depends on the stability and rigidity of the surgical fixation [10], which has generated significant interest pertaining to the value of dual plate fixation versus single plate fixation for added stability. Unfortunately, there is currently limited evidence regarding the impact of dual versus single plate fixation for humerus fractures on rates of nonunion [11], necessitating investigation. However, total complication rates and the added cost of a second plate are considerations that orthopedic surgeons must weigh when potentially choosing dual plate fixation to lower rates of nonunion after operative treatment [6, 11]. As biomechanical data have suggested that dual plate may be superior to single plate fixation when feasible [12, 13], there have been numerous clinical studies comparing dual plate versus single plate fixation in adult patients in the past 10 years to attempt to solve this clinical question [6–8, 11, 14–21]. However, all of these studies were relatively underpowered with small sample sizes and no systematic review and meta-analysis has been conducted on this topic, limiting consensus. Therefore, the purpose of this study was to compare risk of nonunion in adult patients who underwent dual plate versus single plate fixation for humerus fractures to enhance decision-making and improve patient care. Secondarily, this study used subgroup analysis to determine if location of humerus fracture or surgical indication further impacted clinical outcomes.

## Methods

### Study creation and registration

This study is a systematic review and meta-analysis performed under the guidance of the preferred reporting items for systematic reviews and meta-analyses (PRISMA) guidelines [22]. The main literature search was completed using PubMed, CINAHL, MEDLINE, Web of Science, and SPORTDiscus from database inception until January 9th, 2025. Subsequently, a gray literature search using the first fifty search results on google scholar was performed on January 10th, 2025. The search algorithm used in both searches was (dual OR double OR “double-plate”) AND (single OR “single plate”) AND (humerus OR humeral) AND (plate OR plating). This systematic review and meta-analysis were pre-registered prior to data extraction on PROSPERO (CRD42025638571) in order to improve study transparency and quality.

### Inclusion and exclusion criteria

The inclusion criteria for this study were articles that examined clinical outcomes for humerus fractures using dual versus single plate fixation, were comparative in study design, examined adult patients (> 18 years old), were written in English, and were in full-text. The exclusion criteria for this study were articles that were systematic reviews, meta-analyses, case reports, narrative reviews, animal studies, strict biomechanical studies, cadaver studies, had pediatric patients, and did not have full-text in English.

### Article search process

Articles were retrieved and downloaded into Rayyan for ease of screening [23]. Duplicate articles were first removed, followed by a screening process by title and abstract. Next, the remaining articles were screened by full-text for study inclusion. A gray literature search was performed by screening the first fifty results on google scholar, and a full reference search was then undertaken of all of the included articles. This article search process was done by a single author with any disagreements solved by the first author.

### Data extraction process

Data extracted included first author, year of publication, type of study, inclusion and exclusion criteria, information on surgical technique, diagnosis/fracture characteristics, patient demographics (age, male/female, etc.), operative time, estimated blood loss, time until union, nonunion rates, and total complications. Data extraction was done independently by two different authors with any disagreements resolved by discussion between the two authors after independent data extraction and any final decisions on the extracted data were made by the first author. Data were only extracted for information on single plate and dual plate within a study, but not other recorded fixation methods (such as intramedullary nail).

### Primary and secondary outcomes

The primary outcome of this study was the risk of nonunion for distal humerus/humeral shaft fractures. Secondary outcomes included subgroup analysis of nonunion by location and indication, total complications, estimated blood loss, operative time, risk of premature hardware failure, and union time. Articles were too limited and/or heterogeneous for other outcomes, such as patient reported outcomes.

### Article quality assessment

Articles were assessed for quality via the methodological index for non-randomized studies (MINORS) [24]. Grading was done independently by two different authors with any disagreements resolved by discussion. Articles were determined to be “low quality” (< 15 points), “moderate quality” (15–23 points), or “high quality” (24 points) based on the MINORS score as seen elsewhere in the literature [25].

### Certainty assessment

The certainty of evidence was assessed via the grading of recommendations, assessment, development, and evaluation (GRADE) approach [26]. Within the GRADE approach, certainty is determined to be “very low,” “low,” “moderate,” or “high” with evidence from observational studies starting at “low” certainty.

### Statistical analysis

The statistical package for the social sciences (SPSS) version 30.0 was used for statistical analysis in this study. Descriptive statistics were used where appropriate to assist with data presentation. Frequency weighted averages were used for descriptive purposes and represent an aggregate of means or medians weighted by sample size. A random effects meta-analysis using risk ratio (RR) or unstandardized mean difference (MD) with 95% confidence intervals (CI) was used in this study for effect size for binary or continuous variables, respectively. To facilitate a comprehensive meta-analysis, studies with at least one case of a zero event had a continuity correction of 0.5 added to all values in the study by the SPSS software as noted elsewhere [27]. Studies with more events than patients were corrected to equal number of events and patients to allow for meta-analysis statistics, but the actual values were maintained for reporting purposes. For continuous data that were missing standard deviations, we used the standard deviations from other included articles with the most similar data in terms of sample size and means as standard deviation substitution is a method used elsewhere to allow for a more comprehensive meta-analysis [28]. Finally, fragility indices were not used due to the observational nature of the included studies.

## Results

### Initial search and article quality results

**Fig. 1:**
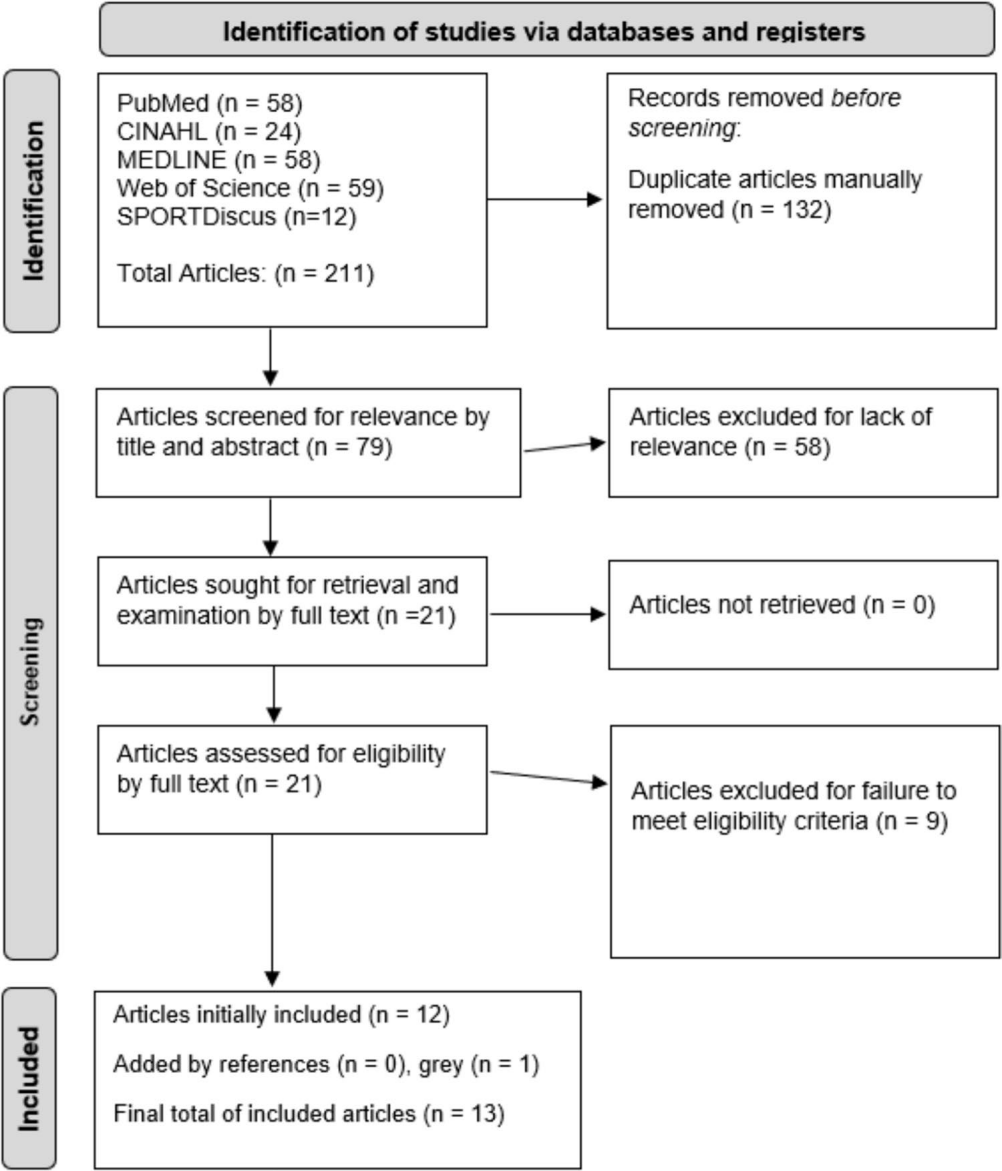
The preferred reporting items for systematic reviews and meta-analyses (PRISMA) diagram

A total of 13 articles were included out of 211 articles initially retrieved (Fig. 1) [6–8, 11, 14–21, 29]. Twelve articles were found via the database search with one article being added via the gray literature. All included articles were retrospective comparative studies with a mean MINORS score of 17.5 ± 1.0 points (Table 1). Furthermore, all articles were determined to be of “moderate quality” based on scoring. Finally, based on article quality and study design, the certainty of evidence for all outcomes was “very low.”

**Table 1:** The methodological index for non-randomized studies (MINORS) scores for all articles

References	Total MINORS score	Clearly stated aim	Inclusion of consecutive patients	Prospective collection of data	End points appropriate to study aim	Unbiased assessment of study end point	Follow-up period appropriate to study aim	Less than 5% lost to follow-up	Prospective calculation of the study size	Adequate control group	Contemporary groups	Baseline equivalence of groups	Adequate statistical analysis
Tanaka [17]	18	2	2	0	2	2	2	2	0	0	2	2	2
Meloy [29]	15	2	2	0	2	2	1	0	0	0	2	2	2
Mao [18]	18	2	2	0	2	2	2	2	0	0	2	2	2
Seo[16]	17	2	2	0	2	2	2	2	0	0	2	1	2
Akdemir [8]	18	2	2	0	2	2	2	2	0	0	2	2	2
Tomori [6]	18	2	2	0	2	2	2	2	0	0	2	2	2
Tosun [7]	18	2	2	0	2	2	2	2	0	0	2	2	2
Karagoz [21]	18	2	2	0	2	2	2	2	0	0	2	2	2
Gaston [11]	18	2	2	0	2	2	2	2	0	0	2	2	2
Unal [14]	18	2	2	0	2	2	2	2	0	0	2	2	2
Tecimel [15]	17	2	2	0	1	2	2	2	0	0	2	2	2
Durgut [20]	18	2	2	0	2	2	2	2	0	0	2	2	2
Ku [19]	16	2	2	0	2	2	2	2	0	0	1	1	2

### Patient characteristics

**Fig. 2:**
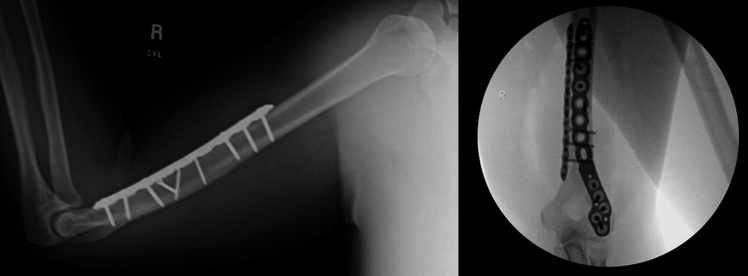
Examples of single plate fixation (left) and dual plate fixation (right) for humeral shaft fractures

Patients (*n* = 788) were 47.8% male (44.9% female; 7.3% unknown) with a frequency weighted average age of 50.4 ± 14.2 years and a frequency weighted average follow-up time of 18.1 ± 14.4 months with 378 patients (48.0%) receiving dual plate and 410 patients (52.0%) receiving single plate fixation (Table 2; Fig. 2). There were 302 distal humerus fractures (38.4%) and 486 humeral shaft fractures (61.7%) in the entire cohort. There were 162 patients (20.6%) from four articles treated for nonunion from a previous humerus fracture and 626 patients (79.4%) from nine articles treated for primary humerus fractures.

**Table 2:** Patient characteristics from all thirteen articles in this systematic review and meta-analysis

References	Inclusion criteria	Exclusion criteria	Indication/location	Group by plate	Fracture type	Patients, *n*	Age (mean (SD) or median (range) (yrs)	Males (%)	Follow-up (mean (SD) or median [IQR] or (range)) (months)
Tanaka [17]	Only AO Type A2 Fracture, 65 + age	Follow-up less than 6 months, numerous fractures, incomplete data, and medial screw fixation alone	Primary fracture/distal humerus	Single plate	–	26	81 (6.86)	7 (26.9)	12 (6–14.75)
				Dual plate	–	63	78.95 (6.74)	16 (25.4)	12 (9.5–16)
Meloy [29]	All patients who had undergone operative fixation for closed distal humeral fracture	–	Primary fracture/distal humerus	Single plate	A2: 5,A3: 43	45	35 (19.4)	22 (48.89)	6.1 (2–24)
				Dual plate	A2: 8, A3: 37	48	43 (22.16)	18 (37.5)	6.1 (2–24)
Mao [18]	Fractures related to arm-wrestling that were treated using open reduction and internal fixation with double or single plating	Under 18 years old, fractures involving articular surface or humeral condyle, presence of numerous fracture sites, follow-up shorter than 1 year, cancer history, ligament injury, and preoperative radial palsy	Primary fracture/distal humerus	Single plate	A1: 13, B1: 3, C1: 2	18	27.56 (6.04)	15 (83.33)	13 (12.25–13.75)
				Dual plate	A1: 12, B1: 3, C1: 1	16	28.88 (6.12)	14 (87.5)	13 (12.25–13.75)
Seo [16]	Humeral shaft fractures classified as 12-A, 12-B, or 12-C according to the classification scheme of the AO/OTA, age > 18 years, and treatment with open reduction and plate fixation	Ipsilateral around elbow injuries, open fracture, and revision cases, including those for nonunion and malunion	Primary fracture/humeral shaft	Single plate	A: 18, B: 16, C: 6	40	43.6 (18.8)	29 (72.5)	13.7 (3.8)
				Dual plate	A: 10, B: 6, C: 4	20	45 (20.5)	11 (55)	9.3 (3.1)
Akdemir [8]	Patients who did not achieve union within six months with conservative or surgical methods and who were followed for at least one year after surgery were included in the study	Pathological fractures, infected cases of nonunion, type 3b or 3c open fractures, patients whose skeletal maturity was not yet complete, and patients with intraarticular extension fractures	Nonunion/humeral shaft	Single plate	–	31	50.52 (17.27)	16 (51.61)	41.05 (20.02)
				Dual plate	–	22	56.73 (15.85)	9 (40.91)	37.97 (31.94)
Tomori [6]	This study included older adults (age > 65 years) with a diagnosis of transcondylar fracture of the humerus, (AO/OTA classification 13A2-3; transverse, transmetaphyseal fracture), and more than 3 months of follow-up data	Pathological fracture, history or elbow trauma, history of elbow OA, or rheumatoid arthritis	Primary fracture/distal humerus	Single plate	–	11	78.5 (6.8)	0 (0)	21.6 (18.6)
				Dual plate	–	17	81.1 (6.9)	2 (11.7)	12.8 (8.8)
Tosun [7]	All the fractures were located between 3 cm distal to surgical neck or 4 cm proximal to the olecranon fossa. Humeral shaft fractures were treated operatively in case of > 20° angulation anteriorly, > 15–30° varus/valgus deformation, > 3 cm shorting, > 20° rotation, failure or poor alignment after casting, accompanying vascular injury (nerve palsy was not a definitive indication), open fractures, polytrauma, and patients’ choice	The patients with pathological fractures, follow-up of shorter than 3 months, patients treated with minimally invasive percutaneous osteosynthesis, and old neglected fractures and refractures were excluded from the study	Primary fracture/humeral shaft	Single plate	A: 31, B: 14, C: 1	46	40.7 (3)	37 (80.43)	3.85 (0.25)
				Dual plate	A: 13, B: 6, C: 5	24	42.9 (3.8)	15 (62.5)	4.45 (0.4)
Karagoz [21]	Patients who underwent surgery for humeral shaft fracture	Non-adults, less than one-year follow-up, tumor-related pathological fractures, and elbow or shoulder injuries	Primary fracture/humeral shaft	Single plate	A: 14, B: 14, C: 3	31	44.61 (19.10)	17 (54.8)	52.87 (24.02)
				Dual plate	A: 11, B; 7, C: 2	20	47.55 (17.97)	12 (60)	44.25 (22.88)
Gaston [11]	Patients receiving surgical treatment for humerus diaphyseal fractures	Under 18 years of age, lost to follow-up, tumor-related pathological fractures, fractures around a periprosthetic implant or previous plate, revision cases for re-injury within 12-month follow-up period and duration of injury date to surgery of over 90 days	Primary fracture/humeral shaft	Single plate	A: 46, B: 21, C: 12	79	44.7 (19.7)	40 (51)	11.88 (10.65)
				Dual plate	A: 40, B: 19, C: 5	64	50.4 (19.7)	22 (34)	9.05 (5.525)
Unal [14]	Patients receiving surgical treatment for humerus diaphyseal nonunion	Less than 12-month follow-up, infected humeral nonunion, and those missing data	Nonunion/humeral shaft	Single plate	–	14	48.14 (16.03)	9 (64.3)	31 (16.9)
				Dual plate	–	17	47.29 (16.24)	14 (82.4)	25.4 (15.6)
Tecimel [15]	Operated on for treatment of humerus shaft fracture nonunion	Pathological fracture, atrophic pseudoarthrosis, open fracture, infected nonunion, fractures requiring soft tissue or vascular reconstruction, chronic disease, limited elbow/shoulder range of motion due to previous surgery, musculoskeletal disease, or missing data	Nonunion/humeral shaft	Single plate	A: 23, B: 6, C: 2	31	53.3 (10.6)	20 (64.5)	40.8 (9.8)
				Dual plate	A: 18, B: 5, C: 2	25	54.5 (8.2)	16 (64)	40.7 (9.6)
Durgut [20]	Patients receiving surgical treatment for humerus diaphyseal nonunion	Patients with infected nonunion, brachial plexus injury, patients treated with intramedullary nailing and Ilizarov external fixator, and patients with diseases that disrupted bone mineralization such as chronic renal failure were excluded from the study	Nonunion/humeral shaft	Single plate	–	11	39 (19–56)	8 (72.73)	23.2 (6–96)
				Dual plate	–	11	43.5 (23–64)	8 (72.73)	13 (6–36)
Ku [19]	Adults who underwent open reduction internal fixation for low transcondylar fractures with follow-up greater than 6 months	AO/OTA type B and C distal humerus fracture; type A distal humerus fracture other than A2.3 of AO/OTA type A; open fracture of Gustilo and Anderson Classification 23; revision open reduction internal fixation due to fixation failure; and loss to follow-up	Primary fracture/distal humerus	Single plate	–	27	–	–	–
				Dual plate	–	31	–	–	–

SD, standard deviation; IQR, interquartile range; OTA, Orthopaedic Trauma Association

### Operative time for dual plate versus single plate fixation

There was no statistically significant difference in operative time for patients who received dual plate (*n* = 161) as compared to patients who received single plate fixation (*n* = 228) via meta-analysis of six articles (*p* = 0.107; MD = 23.90 min [95% CI − 5.20, 53.01]). The frequency weighted mean operative time for dual and single plate was 179.4 ± 85.6 and 143.5 ± 76.9 min, respectively (Table 3).

**Table 3:** Clinical outcomes for dual plate versus single plate fixation from the thirteen included articles in this systematic review and meta-analysis

References	Indication/location	Groups by plate	Patients, *n*	Operative time (median [IQR] or mean (SD)) (minutes)	Estimated blood loss (mean (SD)) (mL)	Time to operation (median [IQR] or mean (SD)) (days)	Time to union (median [IQR] or mean (SD) or (range)) (months)	Total complications, *n* (%)	Nonunion, *n* (%)	Premature hardware failure, *n* (%)
Tanaka [17]	Primary fracture/distal humerus	Single plate	26	119 (103.25–148.5)	–	7.5 (4–10.5)	5.5 [3–6]	2 (7.7)	1 (3.8)	–
		Dual plate	63	179 (140–248.25)	–	6 (4–8.75)	5 [3–6]	32 (50.8)	5 (7.9)	–
Meloy [29]	Primary fracture/distal humerus	Single plate	45	–	–	–	–	3 (6.7)	1 (2.2)	1 (2.2)
		Dual plate	48	–	–	–	–	15 (31.3)	0 (0)	0 (0)
Mao [18]	Primary fracture/distal humerus	Single plate	18	155.56 (35.40)	205.56 (95.32)	–	3 (0.63)	1 (5.5)	0 (0)	–
		Dual plate	16	196.13 (56.7)	293 0.75 (125)	–	3.167 (0.544)	3 (18.8)	0 (0)	–
Seo [16]	Primary fracture/humeral shaft	Single plate	40	84.1 (31.6)	179.4 (89.8)	4.5 (3.6)	–	15 (37.5)	9 (22.5)	2 (5)
		Dual plate	20	71.2 (22.8)	157.8 (46.5)	4.7 (3.9)	–	9 (45)	5 (25)	0 (0)
Akdemir [8]	Nonunion/humeral shaft	Single plate	31	–	–	696.9 (741.9)	4.96 (2.027)	6 (19.4)	3 (9.68)	–
		Dual plate	22	–	–	505.2 (511.2)	5.25 (2.049)	3 (13.6)	2 (9.09)	–
Tomori [6]	Primary fracture/distal humerus	Single plate	11	–	–	21.2 (38.1)	–	12 (100)	0 (0)	3 (27.3)
		Dual plate	17	–	–	17.8 (25.3)	–	11 (64.7)	0 (0)	0 (0)
Tosun [7]	Primary fracture/humeral shaft	Single plate	46	67.2	190.62	–	4.088	5 (10.9)	2 (2.02)	–
		Dual plate	24	71.25	202.5	–	3.6875	3 (12.5)	1 (1.01)	–
Karagoz [21]	Primary fracture/humeral shaft	Single plate	31	82.90 (5.43)	214.68 (22.32)	3.71 (3.20)	–	23 (74.2)	8 (26)	4 (12.9)
		Dual plate	20	90.05 (7.71)	165.50 (10.63)	3.65 (2.28)	–	5 (25)	0 (0)	1 (5)
Gaston [11]	Primary fracture/humeral shaft	Single plate	79	243.1 (62.40)	167.9 (101.5)	8.1 (13.9)	–	12 (15.2)	2 (3)	5 (6.3)
		Dual plate	64	261.5 (67.8)	185.2 (92.1)	6.3 (10.5)	–	11 (17.2)	1 (2)	3 (4.7)
Unal [14]	Nonunion/humeral shaft	Single plate	14	135.3 (27.9)	631.3 (340.3)	246 (102)	31 (16.9)	1 (7.1)	1 (7.1)	–
		Dual plate	17	239.4 (76.1)	836 (544.6)	666 (867)	25.4 (15.6)	7 (41.2)	3 (17.65)	–
Tecimel [15]	Nonunion/humeral shaft	Single plate	31	–	–	–	4.475 (0.5)	5 (16.1)	0 (0)	2 (6.5)
		Dual plate	25	–	–	–	4.55 (0.575)	0 (0)	0 (0)	–
Durgut [20]	Nonunion/humeral shaft	Single plate	11	–	–	–	4.8 (2–11)	1 (9.1)	1 (9.1)	–
		Dual plate	11	–	–	–	3.85 (1.75–8)	0 (0)	0 (0)	–
Ku [19]	Primary fracture/distal humerus	Single plate	27	–	–	–	–	–	2 (7.4)	–
		Dual plate	31	–	–	–	–	–	2 (6.4)	–

SD, standard deviation; IQR, interquartile range

### Estimated blood loss for dual plate versus single plate fixation

There was no statistically significant difference in estimated blood loss between patients who received dual plate (*n* = 161) as compared to patients who received single plate fixation (*n* = 228) via meta-analysis of six articles (*p* = 0.818; MD = 4.76 mL [95% CI − 35.68, 45.20]). The frequency weighted mean estimated blood loss for dual and single plate was 261.4 ± 201.3 mL and 212.3 ± 108.6 mL, respectively (Table 3).

### Risk of nonunion for dual plate versus single plate fixation

**Fig. 3:**
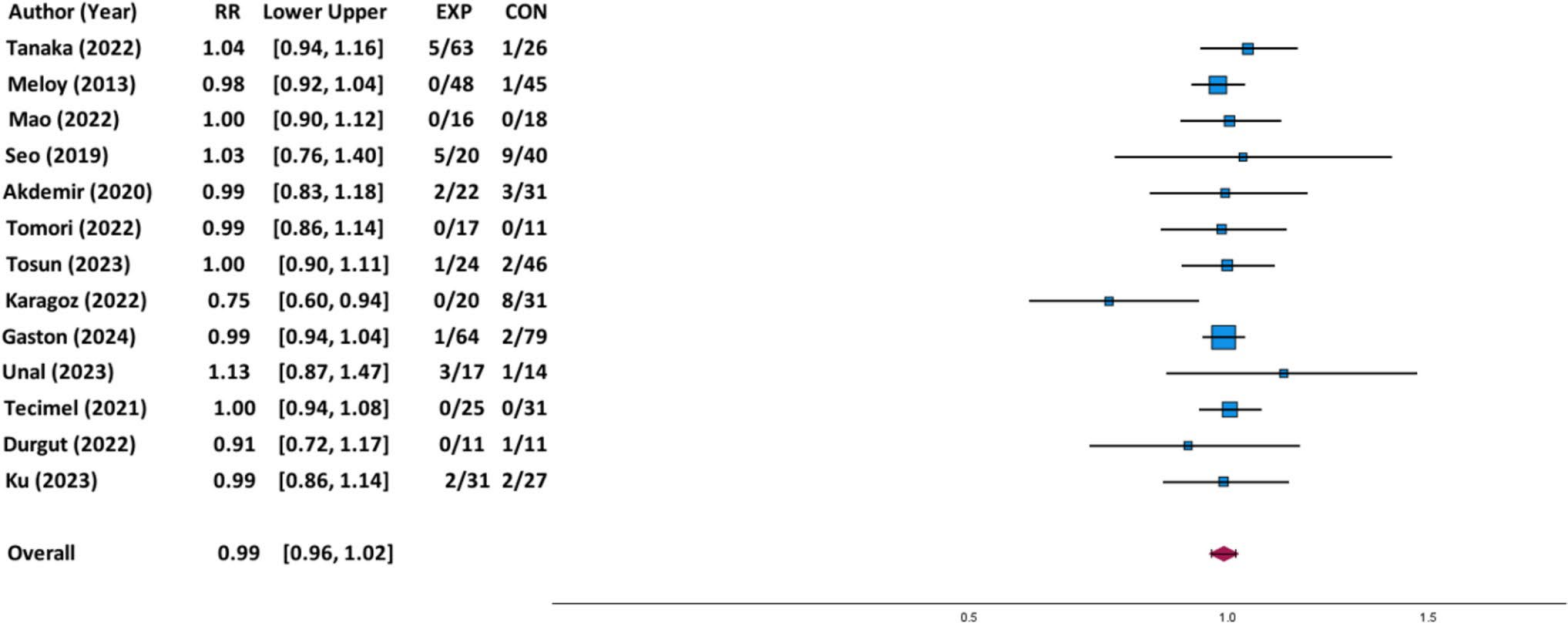
Forest plot for nonunion for dual versus single plate fixation for all studies. Experimental group (EX) refers to dual plate and control group (CON) refers to single plate with data recorded as events/total

There was no statistically significant difference in risk of nonunion between patients who underwent dual plate fixation (*n* = 378) as compared to patients who underwent single plate fixation (*n* = 410) for humerus fractures via meta-analysis of thirteen articles (*p* = 0.502; RR: 0.99 [95% CI 0.96, 1.02]; 5.0% versus 7.3%; Fig. 3). There were 19 cases (5.0%) and 30 cases (7.3%) of nonunion for dual and single plate fixation, respectively (Table 3). For subgroup analysis by location of the humerus fracture, there was no statistically significant difference in risk of nonunion between dual plate versus single plate fixation for distal humerus fractures (*p* = 0.776; RR: 0.99 [95% CI 0.95, 1.04]) or humeral shaft fractures (*p* = 0.524; RR: 0.99 [95% CI 0.96, 1.02]). For subgroup analysis by indication for surgery, there was no statistically significant difference in risk of nonunion between dual plate versus single plate fixation for primary fracture (*p* = 0.426; RR: 0.99 [95% CI 0.96, 1.02]) or nonunion (*p* = 0.928; RR: 1.00 [95% CI 0.94; 1.07]).

### Time until union by dual plate versus single plate fixation

There was no statistically significant difference in time until union among patients who received dual plate (*n* = 115) as compared to patients who received single plate fixation (*n* = 151) for humerus fracture via meta-analysis of six articles, regardless of surgical indication (*p* = 0.332; MD = − 0.21 months [95% CI − 0.64, 0.22]). The frequency weighted mean time until union for patients who received dual plate and single plate fixation was 7.3 ± 7.6 months and 6.8 ± 7.8 months, respectively (Table 3). On subgroup analysis by indication for surgery, there was no statistically significant difference in time until union for adult patients with dual plate fixation versus single plate fixation for primary fracture (*p* = 0.627; MD = − 0.14 months [95% CI − 0.69, 0.42]) or nonunion (*p* = 0.476; MD = − 0.28 months [95% CI − 1.03, 0.48]).

### Risk of total complications by dual plate versus single plate fixation

**Fig. 4:**
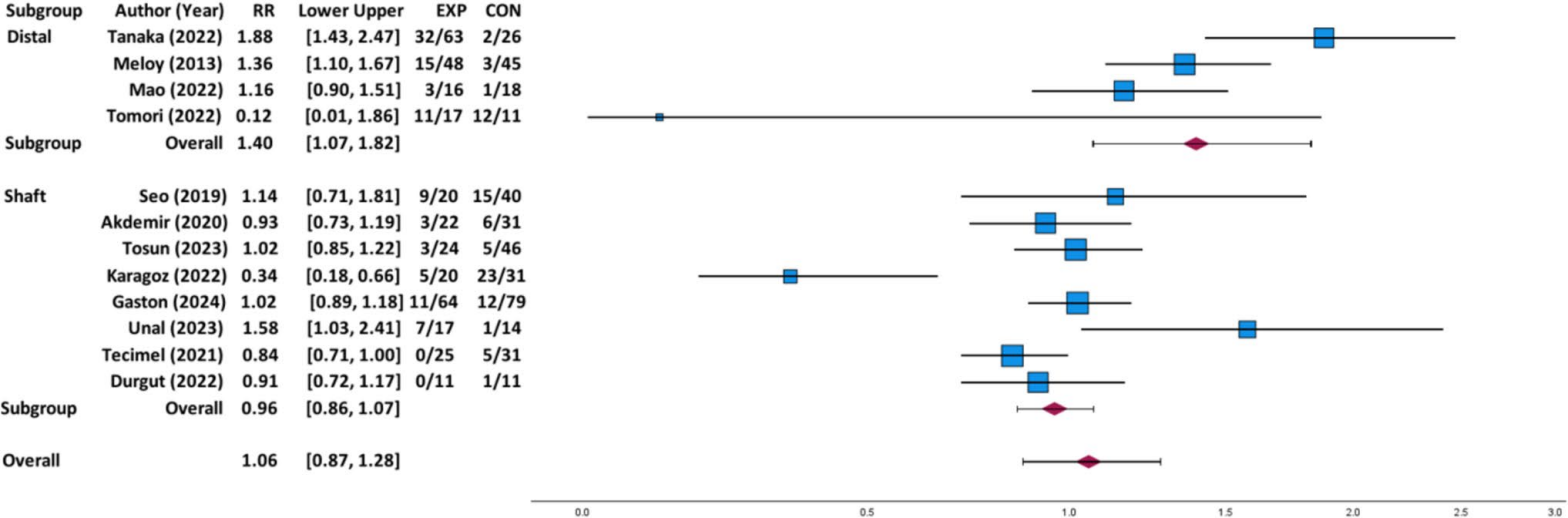
Forest plot for total complications for dual versus single plate fixation with subgroup analysis by location of humerus fracture (Distal = distal humerus fracture and Shaft = humeral shaft fracture). The experimental group (EXP) refers to dual plate fixation, and control group (CON) refers to single plate fixation in the plot. The actual values from each study are recorded as events/total for the two groups

There was no statistically significant difference in total complications between patients who underwent dual plate fixation (*n* = 347) as compared to patients who underwent single plate fixation (*n* = 383) for humerus fractures (*p* = 0.575; RR: 1.06 [95% CI 0.87, 1.28]; Fig. 4). There are 99 total complications (28.5%) and 86 total complications (22.5%) for patients with dual and single plate fixation, respectively.

While there was no statistically significant difference for humeral shaft fractures (*p* = 0.457; RR: 0.96 [95% CI 0.86, 1.07]), there was a statistically significant increase in total complications among patients who received dual plate fixation as compared to patients who received single plate fixation for distal humerus fractures (*p* = 0.014; RR: 1.40 [95% CI 1.07, 1.82]). There are 61 total complications out of 144 patients (42.4%) and 18 total complications out of 100 patients (18.0%) for patients with dual and single plate fixation, respectively. For subgroup analysis by indication for surgery, there was no statistically significant difference in total complications between dual plate versus single plate fixation for primary fracture (*p* = 0.715; RR: 1.06 [95% CI 0.78, 1.44]) or nonunion (*p* = 0.748; RR: 0.97 [95% CI 0.80; 1.18]).

### Risk of premature hardware failure for dual plate versus single plate fixation

There was no statistically significant difference in risk of premature hardware failure between patients who received dual plate (*n* = 169) versus single plate (*n* = 206) for primary humerus fractures via meta-analysis of five articles (*p* = 0.139; RR: 0.97 [95% CI 0.93, 1.01]). There were four cases (2.4%) and 15 cases (7.3%) of premature hardware failure for dual plate and single plate fixation, respectively (Table 3).

## Discussion

This study represents the first systematic review and meta-analysis examining dual plate fixation versus single plate fixation for humeral shaft and distal humerus fractures in adult patients. The results of this study cautiously call into question the widespread value of dual plate fixation as this study found no statistically significant decrease in risk of nonunion (*p* = 0.502; RR: 0.99), risk of premature hardware failure (*p* = 0.139; RR: 0.97), and risk of total complications (*p* = 0.575; RR: 1.06) with no statistically significant difference in time until union (*p* = 0.332; MD = − 0.21 months). These associations were upheld during subgroup analyses as there was no statistically significant decrease in risk of nonunion when dual plate fixation was compared to single plate fixation for distal humerus fractures (*p* = 0.776; RR: 0.99), humeral shaft fractures (*p* = 0.524; RR: 0.99), for primary fracture (*p* = 0.426; RR: 0.99), or nonunion of prior fracture (*p* = 0.928; RR: 1.00). Additionally, there was no statistically significant risk of total complications in all subgroups (*p* > 0.05), except for distal humerus fractures (*p* = 0.014; RR: 1.40), although this association should be taken with caution. Overall, the results of this study appear to carefully call into question dual plate fixation, unless required by indication, as it may not provide meaningful benefits in terms of bony union.

An additional consideration in this debate is the impact of dual plate versus single plate fixation on patient reported outcome measures, such as the quick disabilities of the arm, shoulder, and hand (Quick DASH). While study heterogeneity prevented meta-analysis on patient reported outcome measures, multiple studies found no statistically significant difference in Quick DASH scores for dual plate versus single plate fixation for humerus fractures [8, 14, 15, 21]. However, Mao et al. [18] found dual plate fixation to have higher American shoulder and elbow surgeons (ASES) scores at 2 weeks (*p* < 0.001), 1 month (*p* = 0.002), and three months (*p* = 0.029), but not at the final follow-up of 1 year (*p* = 0.13) in comparison with single plate fixation for primary distal humerus fractures [18]. Similarly, several articles found no statistically significant difference in mayo elbow performance scores (MEPS) at final follow-up between dual and single plate fixation for humerus fractures [6, 15, 17], but Tecimel et al. [15] did report higher scores for the MEPS among patients who received dual plate fixation at 3-month postoperative follow-up [15].

Importantly, the impact of dual plate versus single plate fixation regarding elbow range of motion (ROM) represents another component involved in surgical decision-making. Although study heterogeneity once again precluded meta-analysis on ROM outcomes, Tanaka et al. [17] found no statistically significant difference in total elbow ROM at 3 months (*p* = 0.182), 6 months (*p* = 0.487), and final follow-up (*p* = 0.544) for dual plate versus single plate fixation for primary distal humeral fractures [17]. Contrarily, Meloy et al.^29^ found that single plate fixation had statistically significant higher elbow flexion and elbow extension ROM as compared to dual plate fixation for primary distal humeral fractures [29]. Given these conflicting results, future research is needed to elucidate the relationship between dual plate versus single plate fixation and elbow ROM. Finally, cost and hardware availability remain an important part of this debate [30], especially given that the observation that dual plating may not provide clinically meaningful improvements in bony union. Future studies should explore the relevant cost metrics at various time points to further enhance decision-making.

Strengths of this study include the largest sample size to date exploring dual plate versus single plate fixation for humeral shaft and distal humerus fractures in adults, a robust set of subgroup analyses by fracture location and surgical indication, and a vigorous assessment of the current literature. However, there are numerous limitations of this study that necessitate caution and consideration. First, there are currently no prospective non-randomized or randomized controlled trials on this topic, limiting this systematic review and meta-analysis to retrospective studies. However, we attempted to mitigate this limitation by including only comparative studies, using a random effects meta-analysis with numerous strategies to provide a comprehensive evaluation, and using the GRADE approach to categorize the certainty of evidence of this study as “very low” for every outcome. Additionally, there is likely substantial heterogeneity between the articles, even within each subgroup analysis, that could potentially skew the results, such as differences in fracture patterns, patient comorbidities, smoking habits, plate placement, and decision-making for the use of dual plate versus single plate fixation. Compounding this heterogeneity is the limited number of available studies, resulting in some degree of underpowered analysis that may have contributed to certain outcomes failing to reach statistical significance. For example, operative time exhibited a non-significant trend toward favoring shorter durations with single plate fixation. Though this finding may seem intuitive from a surgical standpoint, the absence of such statistical significance should not be viewed as definitive, but rather as an illumination of the need for more adequately powered, focused studies. Therefore, surgeons should incorporate the results of this study into their decision-making process with caution, while continuing to select dual plate or single plate fixation on a case-by-case basis. Future research studies should be prospective or randomized in study design, should focus on either distal humerus or humeral shaft fractures, report fracture patterns and/or classifications, and examine nonunion in addition to other outcomes not assessed in this study due to study heterogeneity, such as postoperative elbow ROM and patient reported outcome measures.

## Conclusion

Compared to single plate fixation for humeral shaft and distal humerus fractures in adults, dual plate fixation was not associated with a statistically significant decrease in risk of nonunion, risk of premature hardware failure, and risk of total complications with no statistically significant difference in time until union, estimated blood loss, and operative time based on thirteen moderate quality retrospective studies. In the subgroup analysis for risk of nonunion, there was no statistically significant decrease in risk of nonunion by location (distal humerus fracture or humeral shaft fracture) or indication (primary fracture or nonunion of prior fracture) for dual plate fixation versus single plate fixation. The results of this study may cautiously suggest that dual plate fixation, unless indicated due to fracture comminution or other surgical considerations on a case-by-case basis, may not provide meaningful benefits in terms of bony union in comparison with single plate fixation for distal humerus and humeral shaft fractures. As this study represents the first systematic review and meta-analysis to examine dual plate fixation versus single plate fixation for humeral shaft and distal humerus fractures in adults, caution must be exercised due to the very low certainty of evidence, the retrospective nature of the included studies, and potential low study power.

## Data Availability

No datasets were generated or analysed during the current study.
