# Correction: Risk of nonunion for dual plate versus single plate fixation for humeral shaft and distal humerus fractures: a systematic review and meta-analysis

**DOI:** 10.1007/s00590-025-04539-3

**Published:** 2025-10-07

**Authors:** Anthony N. Baumann, Grayson M. Talaski, Shahabeddin Yazdanpanah, Timothy Guthrie, Tyler Sanda, Michael Makowski

**Affiliations:** 1 College of Medicine, Northeast Ohio Medical University, Rootstown, USA; 2 Department of Orthopaedic Surgery, University of Iowa, Iowa City, USA; 3 Department of Orthopaedic Surgery, Cleveland Clinic Akron General, Cleveland, USA; 4 Department of Rehabilitation Services, University Hospitals, Cleveland, USA

**Correction to: European Journal of Orthopaedic Surgery & Traumatology (2025) 35:372** 10.1007/s00590-025-04491-2

In the original version of this article, Shahabeddin Yazdanpanah was incorrectly denoted as the corresponding author but it should have been Anthony N. Baumann.

The affiliation details for Anthony N. Baumann were incorrectly given as

College of Medicine, Northeast Ohio Medical University, Rootstown, USA and Department of Rehabilitation Services, University Hospitals, Cleveland, USA.

but should have been

Department of Orthopaedic Surgery, Cleveland Clinic Akron General, Cleveland, USA and Department of Rehabilitation Services, University Hospitals, Cleveland, USA.

The original article has been corrected.

